# Efficacy and Safety of Plastic Wrap for Prevention of Hypothermia after Birth and during NICU in Preterm Infants: A Systematic Review and Meta-Analysis

**DOI:** 10.1371/journal.pone.0156960

**Published:** 2016-06-09

**Authors:** Shaojun Li, Pengfei Guo, Qing Zou, Fuxiang He, Feng Xu, Liping Tan

**Affiliations:** 1 Department of Emergency, Children's Hospital of Chongqing Medical University, Chongqing, China; 2 Ministry of Education Key Laboratory of Child Development and Disorders, Chongqing, China; 3 Key Laboratory of Pediatrics in Chongqing, Chongqing, China; 4 Chongqing International Science and Technology Cooperation Center for Child Development and Disorders, Chongqing, China; Hôpital Robert Debré, FRANCE

## Abstract

**Objective:**

This meta-analysis aimed to investigate the efficacy and safety of plastic wrap applied after birth and during NICU in preterm infants for prevention of heat loss in preterm infants.

**Study Methods:**

The Medline (1950 to August 2015), the Cochrane Central Register of Controlled Trials (CENTRAL, Issue 7, 2015), CINAHL (1982 to August 2015) and the Embase (1974 to August 2015) databases were searched for randomized controlled trials (RCTs) or quasi-RCTs with main outcomes related to the core temperature (baseline temperature and/or post-stabilization temperature), hypothermia, mortality rate and hyperthermia.

**Result:**

The included studies were of low to moderate quality. Compared with unwrapped infants, plastic wrap was associated with a significantly higher baseline temperature and post-stabilization temperature both in infants < 28 weeks of gestation (mean difference [MD] = 0.62, 95% CI 0.38 to 0.85; MD = 0.41, 95% CI 0.33 to 0.50, respectively), and in infants between 28 to 34 weeks of gestation (MD = 0.54, 95% CI 0.21 to 0.87; MD = 0.64, 95% CI 0.45 to 0.82, respectively). Use of plastic wrap was associated with lower incidence of hypothermia (relative risk [RR] = 0.70, 95% CI 0.63 to 0.78). However, use of plastic wrap in preterm infants was not associated with decrease in mortality (RR: 0.88, 95% CI 0.70 to 1.12, *P* = 0.31). Incidence of hyperthermia was significantly higher in the plastic wrap group as compared to that in the control group (RR = 2.55, 95% CI: 1.56 to 4.15, *P* = 0.0002). Hyperthermia in the plastic wrap group was resolved within one or two hours after unwrapping the babies.

**Conclusion:**

Plastic wrap can be considered an effective and safe additional intervention to prevent hypothermia in preterm infants. However, its cost-effectiveness and long-term effect on mortality needs to be ascertained by conducting well-designed studies with longer follow-up period.

## Introduction

Hypothermia is known to be an independent risk factor for neonatal mortality in both developed and developing settings.[1–4] Complications associated with neonatal hypothermia include infection, acidosis, coagulation disorders, and respiratory distress syndrome.[5, 6] Early interventions to prevent neonatal hypothermia are thus of vital import. Current practices that are part of routine thermal care such as maintenance of a warm temperature in the delivery room, drying of neonate, use of pre-warmed blanket, radiant warmers or incubators are often inadequate in preventing heat loss in preterm infants.[2, 3] Although infants are kept warm by radiation, potential heat losses can also occur through convection and evaporation. The application of plastic wrap immediately after birth can reduce immediate postnatal evaporative heat loss.

Previous studies have shown that routine thermal care is particularly inadequate for preterm infants owing to their greater vulnerability to heat loss. Reducing heat loss in preterm infants in the first few days after birth has been reported to increase survival rates.[7, 8] The International Liaison Committee on Resuscitation (ILCOR) consensus statement recommends the use of plastic wrap as a standard technique to maintain body temperature.[9] However, the use of plastic wrap in the preterm infants requires further investigation.

A systematic review by McCall EM *et al*. revealed that plastic wraps or bags keep preterm infants warmer leading to higher temperatures on admission to neonatal units and less hypothermia. [10] However, the studies included in the review had a relatively small numbers of infants, and no long-term follow-up. Hence, firm recommendations for clinical practice cannot be based on the review by McCall *et al*. Currently, there is insufficient evidence to suggest a reduction in in-hospital death with the application of plastic wrap.[1] Further, a large randomized controlled trial found no significant differences in mortality between wrapped and unwrapped infants.[11] The aim of this study was to combine the current evidence from eligible RCTs to further systematically evaluate the efficacy and safety of plastic wrap versus conventional thermal care in the prevention of hypothermia in preterm infants.

## Methods

Registered in PROSPERO: CRD42015025397.

A literature search for relevant studies was performed in MEDLINE (reference period: 1950 to August, 2015), the Cochrane Central Register of Controlled Trials (CENTRAL, The Cochrane Library, Issue 7, 2015), EMBASE (1974 to August 2015) and CINAHL (1982 to August 2015) was performed. The key words used were: “low birth weight” and “premature” or “preterm” and “plastic barrier” or “plastic wrap” or “polyethylene” or “plastic bag”, with restrictions of children and clinical trials. The reference lists in the retrieved publications were manually searched to identify additional relevant studies. Two reviewers independently screened all abstracts for eligibility. In the event of any disagreement, a consensus was reached by discussion.

Inclusion criteria were: (1) RCT or quasi-RCT study; (2) study population of preterm infants (< 37 weeks gestational age); (3) intervention: plastic wraps used immediately after birth and/or after during the neonatal intensive care unit (NICU); involved use of transparent plastic wraps and bags made of low density polyethylene or linear low density polyethylene or polyvinylidene chloride; (4) the control intervention comprising of any form of routine thermal care; (5) Infants’ temperatures were measured on admission to the NICU (baseline temperature), and/or after having been stabilized in the NICU (post-stabilization temperature). The core temperature was accessed as continuous and/or dichotomous variables. Axillary temperature is preferred over rectal (core) temperature for routine measurement of temperature, according to the recommendation of the World Health Organization. Hypothermia was defined as a rectal temperature or a axillary temperature of < 36.5°C in intervention and the control groups; (6) secondary outcomes: included mortality rate (death within seven days, death within 28 days and/or death during hospital stay), hyperthermia (defined by an admission temperature to NICU or during the NICU stay ≥ 38.0°C), infection (defined by a culture of pathogenic bacteria from normally sterile body tissue or fluid within seven days of birth) and skin maceration (attributed to the intervention within the first week of birth). Exclusion criteria included: (1) Infants with major congenital malformations, especially those with abdominal wall defects; (2) treatment arm with < 10 patients; (3) studies published in non-English language.

Two reviewers (Shaojun Li, Liping Tan) independently assessed the methodological quality using the Cochrane risk of bias tool. Data was extracted using a structured form that included author details, year of publication, sample size, study setting, interventions and outcomes. Any disagreement was resolved through consensus, or was referred to a third person (Feng Xu).

The meta-analysis was conducted using Review Manager Software (RevMan 5.3) obtained from the Cochrane Collaboration. The Mantel-Haenszel method was followed to estimate relative risk (RR) and risk difference (RD). For quantitative data, the inverse variance method was employed. Subgroup analysis disaggregated by the degree of severity of hypothermia was performed. Relative risk (RR) with 95% confidence interval (CI) was calculated for dichotomous outcomes. Mean difference (MD) with 95% confidence interval was calculated for continuous variables. The treatment effect of individual trials was estimated; heterogeneity among the included trials was assessed by constructing forest plots; the impact of heterogeneity was quantified using the Cochrane Q test and the I^2^ statistic. *P* < 0.1 or I^2^ > 50% was considered indicative of a significant heterogeneity.

In case of significant heterogeneity, the random effects model was employed for data analysis; while the fixed effect model was used in the absence of significant heterogeneity. In the event of statistical heterogeneity, the possible contributors were assessed on sensitivity analyses based on the risk of bias assessment or by the differences in the intervention or outcomes. Publication bias was estimated by Egger’s test and Begg’s test using Stata software (version 11.0, Stata Corporation, College Station, TX, USA).

## Results

A total of 1858 articles were retrieved on the initial literature search, of which 869 were retrieved from PubMed, 416 from Embase, 557 from CINAHL and 16 from the Cochrane Library. A total of 236 studies were eliminated because of duplication, using Endnote software. After screening of the titles and abstracts, 1606 studies were excluded, and the remaining 16 full-text articles were assessed for eligibility. Three articles [12–14] were excluded due to incomplete data. Two articles [15, 16] were excluded due to ineligible study design (S1 Table).

**Fig 1:**
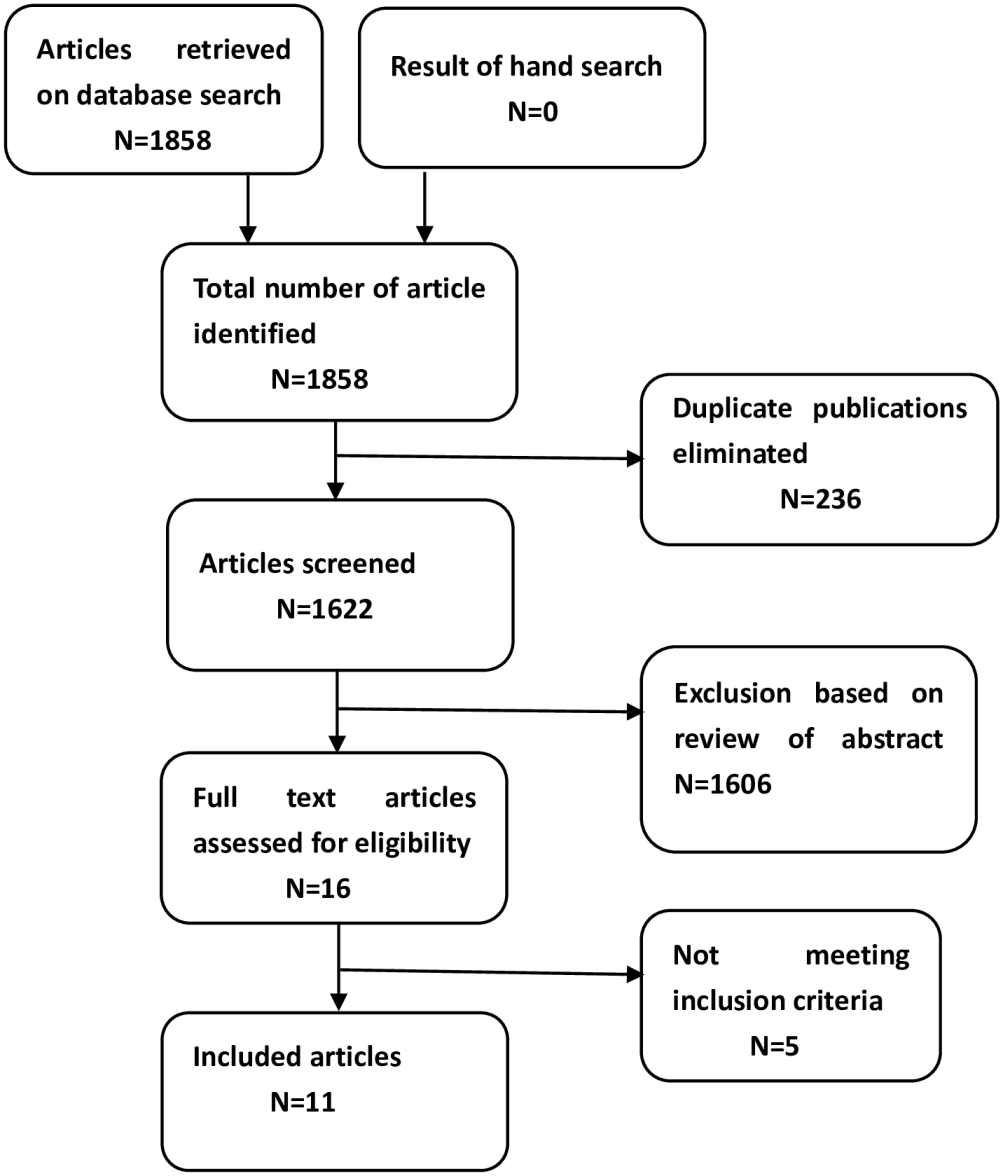
Schematic illustration of literature search and study selection for meta-analysis.

In all, 11 articles [11, 17–26] involving 1601 infants were included in the meta-analysis (Fig 1). Nine studies [11, 17, 18, 20–25] were performed on preterm infants with gestational age < 28 weeks, while 5 studies [19, 20, 22, 25, 26] included those with gestational age < 34 weeks. The main outcome, i.e., core body temperature was reported as a continuous variable in all 11 studies, and as a dichotomous variable (presence of hypothermia) in 7 studies.[11, 17, 18, 20, 21, 23, 26] Mortality (secondary outcome) was reported in 8 studies [11, 17, 18, 20, 21, 23–25], while none of the studies reported data on neurodevelopmental outcomes. Eight studies had reported on the safety aspects (Table 1). [11, 17–21, 23, 24]

**Table 1:** Descriptive characteristics of studies included in the meta-analysis.

Study	Country	Cases (N)	Gestation (weeks)	Birth weight (g)	Treatment Group	Control Group	Definition of hypothermia	Study outcomes	Duration of follow-up	Attrition (%)
Vohra 1999 [25]	Canada	59	< 32	600 to 1200	Polyethylene wrap	conventional care	rectal temperature	mortality, baseline temperature	during NICU admission	3 (4%)
Vohra 2004 [24]	Canada	53	< 28	600 to 1200	Polyethylene wrap	conventional care	rectal temperature	mortality, baseline and poststabilization temperature	before hospital discharge	3 (5%)
Knobel 2005 [23]	USA	88	< 29	600 to 1200	Polyurethane bag	conventional care	rectal temperature < 36.4°C	mortality, baseline temperature, hypothermia	before hospital discharge	0 (0%)
Trevisanuto 2010 [21]	Italy	96	24 to 28	600 to 1200	Polyethylene cap or wrap	pre-warmed towels	axillary temperature < 36.4°C	mortality, baseline and poststabilization temperature, hypothermia	before hospital discharge	21 (18)
Chantaroj 2011 [26]	Thailand	38	< 32	data not available	Polyethylene bag	standard thermal care	rectal temperature < 36.5°C	baseline temperature, hypothermia	during NICU admission	0 (0%)
Rohana 2011 [20]	Malaysia	110	24 to 34	800 to 1800	Polyethylene plastic sheets	warmed towels	axillary temperature < 36.5°C	mortality, baseline and poststabilization temperature, hypothermia	during NICU admission	5 (4%)
Doglioni 2014 [17]	Italy	100	24 to 28	600 to 1200	Polyethylene occlusive wrap	covered only the body	axillary temperature < 36.5°C	mortality, baseline temperature, hypothermia	before hospital discharge	0 (0%)
Maureen 2015 [11]	Canada	801	24 to 28	600 to 1020	Polyethylene occlusive wrap	conventional method	axillary temperature < 36.5°C	mortality, baseline and poststabilization temperature, hypothermia	before hospital discharge or 6 months' corrected age	12 (1.4%)
Smith 2013 [18]	Australia	92	less than 29	500 to 1800	Wrapped with NeoWrap	radiant warmer	axillary temperature < 36.5°C	mortality, baseline and poststabilization temperature	during NICU admission	3(3%)
Leadford 2013 [19]	USA	104	26–34	1000 to 2500	Polyethylene bag	standard thermal care	axillary temperature less than 36.5°C	baseline temperature	during NICU admission	0(0%)
Duman 2006 [22]	Turkey	60	24 to 34	500 to 1500	Polyethylene wrap	conventional care	axillary temperature less than 36.5°C	poststabilization temperature	undescription	0(0%)

### Quality assessment of the included studies

**Fig 2:**
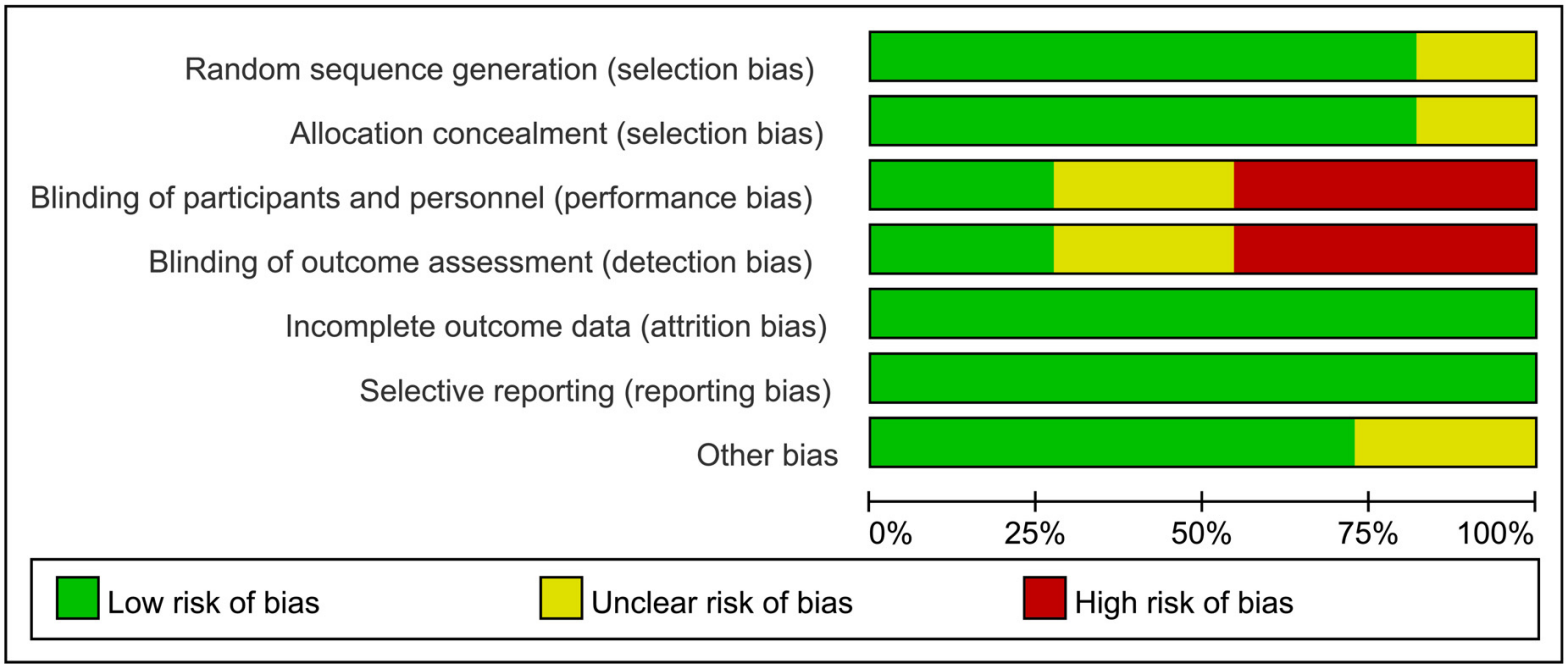
Risk of bias graph.

**Fig 3:**
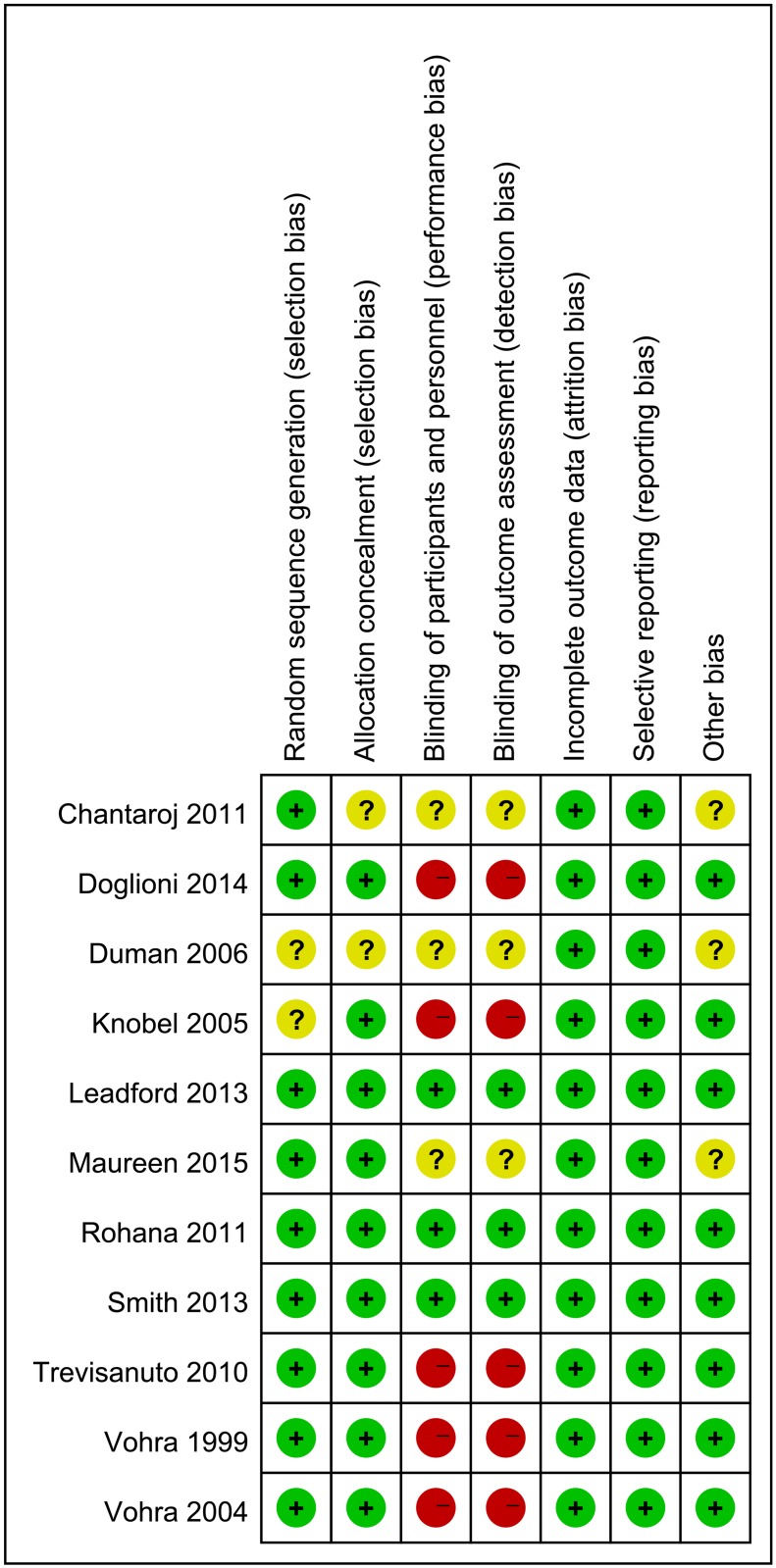
Risk of bias summary.

Figs 2 and 3 show the results of the assessment of risk of bias. All 11 studies were RCTs, with the details on blinding being reported in 9 studies.[11, 17–21, 23, 24] The random sequence generation and allocation were found to be low risk in 9 studies [11, 17–21, 24–26] and unclear in 2 studies.[22, 23] The risk of performance and detection bias was high or unclear in 8 RCTs.[11, 17, 21–26] Follow-up was complete in 5 studies[17, 19, 22, 23, 26]; while the remaining 6 studies had a considerable dropout rate[11, 18, 20, 21, 24, 25], with 3 to 12 (1.4% to 5%) infants not completing the trial. There was insufficient information available to assess whether these studies were free from selective reporting.

### Effect on core body temperature

**Fig 4:**
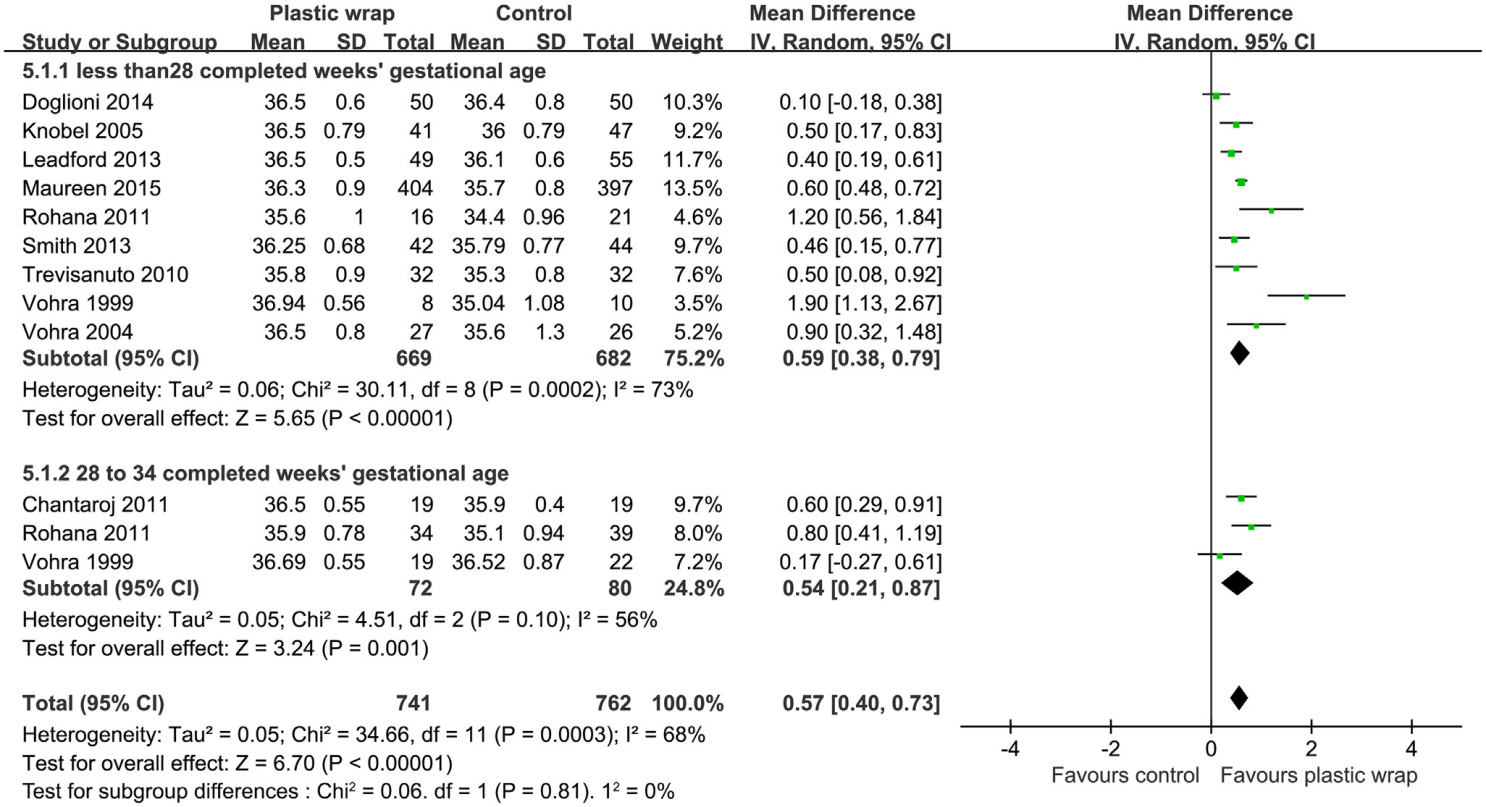
Forest plot showing the effect of plastic wrap versus control intervention on the baseline temperature of preterm infants less than 28 weeks of gestation and at 28 to 34 weeks of gestation. CI, Confidence interval.

**Fig 5:**
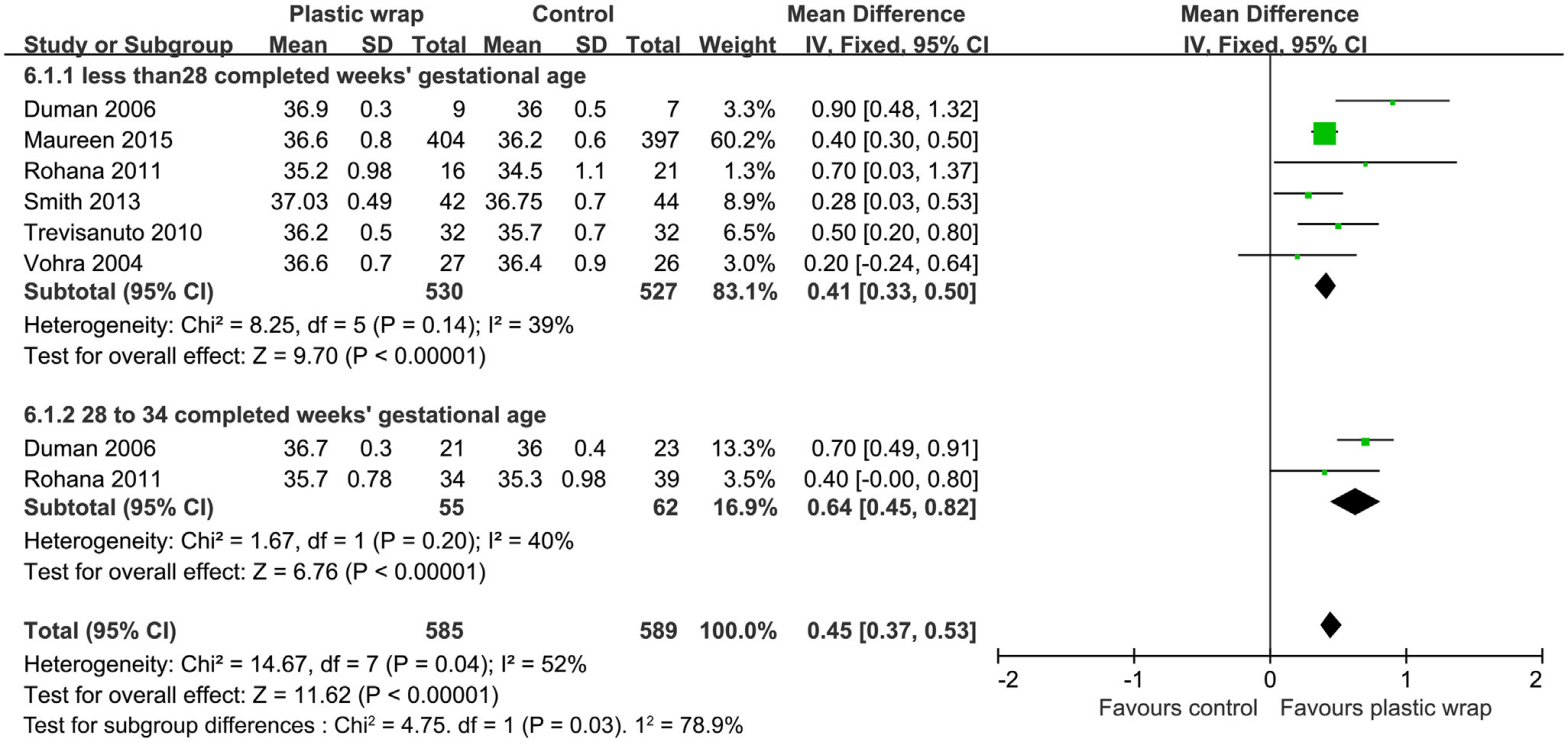
Forest plot showing the effect of plastic wrap versus control intervention on the post-stabilization temperature of preterm infants less than 28 weeks of gestation at 28 to 34 weeks of gestation. CI, Confidence interval.

Nine RCTs [11, 17–25] with a combined subject population of 1307 infants with gestational age < 28 weeks had reported data on baseline temperature and six studies [11, 18, 20–22, 24] with 1057 infants of gestational age < 28 weeks had reported data on post-stabilization temperature. On pooled analysis, the use of plastic wrap was associated with a higher temperature as compared to that achieved with routine thermal care both on baseline temperature (MD = 0.59, 95% CI 0.38 to 0.79) and on post-stabilization temperature (MD = 0.41, 95% CI 0.33 to 0.50) (Figs 4 and 5). The random effect model was used for baseline temperature and fixed effect model was used for the post-stabilization temperature (I^2^ = 73%, *P* = 0.0001; and I^2^ = 39%, *P* = 0.14, respectively). With infants of gestational age between 28 and 34 completed weeks, meta-analysis of three studies [20, 25, 26] on baseline temperature (N = 152) and two studies [20, 22] on post-stabilization temperature (N = 117) also revealed similar results (MD = 0.54, 95% CI 0.21 to 0.87; MD = 0.64, 95% CI 0.45 to 0.82, respectively) (Figs 4 and 5). The random effect model was used for baseline temperature and fixed effect model was used for post-stabilization temperature (I^2^ = 56%, *P* = 0.10; and I^2^ = 40%, *P* = 0.20, respectively).

### Incidence of hypothermia

**Fig 6:**
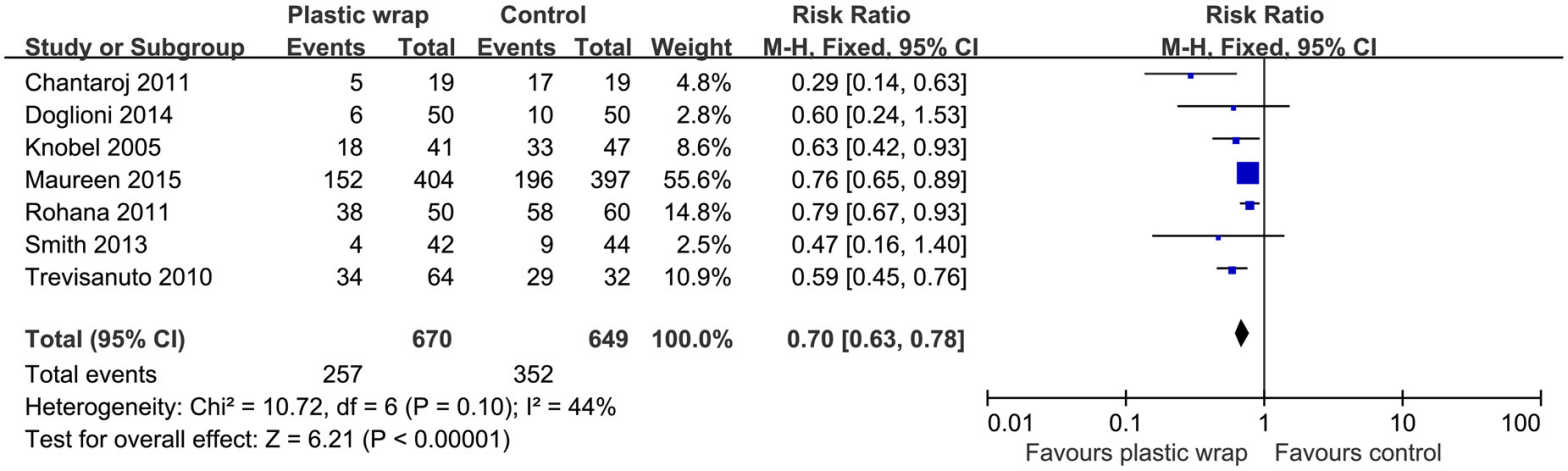
Forest plot showing the effect of plastic wrap versus control intervention on hypothermia of preterm infants. M-H, Mantel-Haenszel; CI, confidence interval.

Seven studies had reported data on the incidence of hypothermia. [11, 17, 18, 20, 21, 23, 26] On meta-analysis, use of plastic wrap was associated with a decreased incidence of hypothermia as compared to that achieved with routine thermal care (RR = 0.70, 95% CI 0.63 to 0.78) (Fig 6). The studies included in the meta-analysis had no significant heterogeneity and the fixed effect model was used (I^2^ = 44%, *P* = 0.10). Only one study[11] had reported degrees of severity of hypothermia (the definition of mild, moderate and severe hypothermia were 35.5–36.5°C, 34.5–35.4°C and < 34.5°C, respectively).

### Effect on mortality

**Fig 7:**
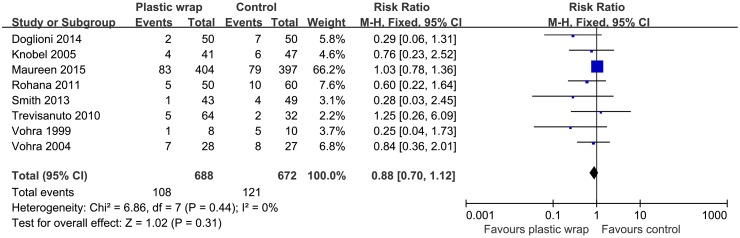
Forest plot showing the effect of plastic wrap versus control intervention on mortality rates of preterm infants. M-H, Mantel-Haenszel; CI, confidence interval.

Eight studies [11, 17, 18, 20, 21, 23–25] had reported data on mortality rate at the end of the follow-up. Pooled analyses of data from these studies showed no significant inter-group difference in the mortality rate between the plastic wrap and the control groups (RR: 0.88, 95% CI: 0.70 to 1.12, *P* = 0.31) (Fig 7). The fixed effect model was used for the analysis, and no significant heterogeneity was observed among these studies (I^2^ = 0, *P* = 0.44).

### Adverse events

**Fig 8:**
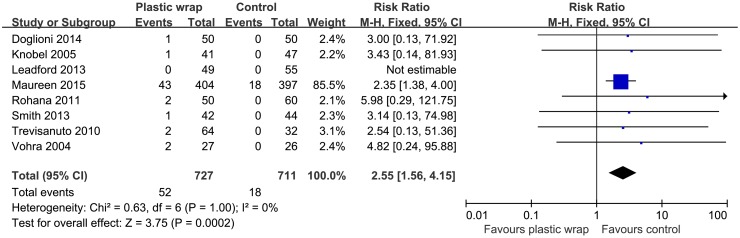
Forest plot showing the effect of plastic wrap versus control intervention on hyperthermia of preterm infants. M-H, Mantel-Haenszel; CI, confidence interval.

Eight studies [11, 17–21, 23, 24] reported data on the incidence of hyperthermia. Incidence of hyperthermia was significantly higher in the plastic wrap group as compared to that in the control group (RR = 2.55, 95% CI: 1.56 to 4.15, *P* = 0.0002) (Fig 8). No significant heterogeneity was observed among these studies using the fixed effect model (I^2^ = 0, P = 1). Descriptive data on one infant each reported by Doglioni N *et al*. [17], Knobel RB *et al*. [23], and Smith J *et al*. [18], and on two infants reported by Vohra S *et al*. [24], indicated that hyperthermia in the plastic wrap group was readily resolved within one or two hours after promptly unwrapping the babies. Data on other adverse effects, such as infection, and skin maceration were not reported in any of the studies, neither was any data available on serious adverse effects.

### Sensitivity analyses

**Fig 9:**
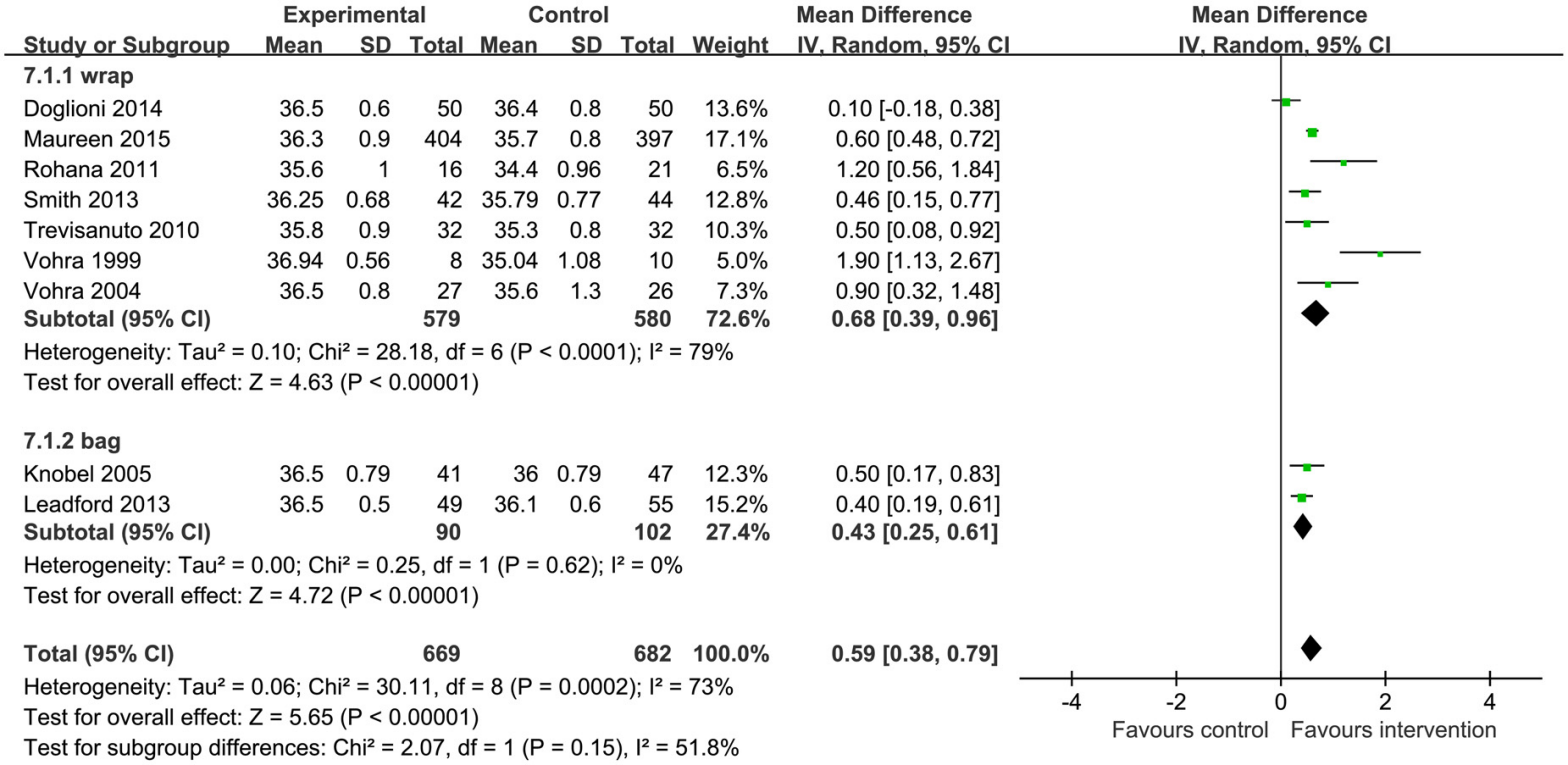
Subgroup analysis of different intervention (wrap or bag) on the baseline temperature of preterm infants less than 28 weeks of gestation. M-H, Mantel-Haenszel; CI, confidence interval.

Based on the risk of bias in blinding, we used the random effect model for baseline temperatures in infants less than 28 weeks of gestational age. After exclusion of five studies [17, 21, 23–25], results of the sensitivity analysis were consistent with the original results (MD = 0.55, 95% CI: 0.37 to 0.74), with an equivalent heterogeneity by use of random effect model (I^2^ = 57%, *P* = 0.08) (Table 2). According to the differences in the intervention (wrap or bag), subgroup analysis using the random effect model for the studies reporting baseline temperature in infants with gestational age less than 28 weeks, showed similar effect size (MD = 0.68, 95% CI: 0.39 to 0.96, MD = 0.43, 95% CI: 0.25 to 0.61, respectively), with a unequal heterogeneity (I^2^ = 79%, *P* < 0.0001, I^2^ = 0%, *P* = 0.62) (Fig 9).

**Table 2:** Sensitivity analyses of baseline temperatures in infants less than 28 weeks of gestational age.

Basis of sensitivity analysis	Overall effect (n = number of studies)	Result of sensitivity analysis (n = number of studies)
Risk of bias assessment(blinding)	MD = 0.59, 95% CI: 0.38 to 0.79; I^2^ = 73%, *P* = 0.0002 (9)	MD = 0.55, 95% CI: 0.37 to 0.74; I^2^ = 57%, *P* = 0.08 (4)

### Publication bias

Studies reporting data on core body temperature of infants with gestational age < 28 weeks were assessed for publication bias (Egger’s test, *P* = 0.200, Begg’s test, *P* = 0.029). The statistical testing revealed an apparent difference that suggested the presence of a potential publication bias, a language bias, and inflated estimates by a flawed methodological design in smaller studies, and/or a lack of publication of small trials with contradictory results.

## Discussion

Our meta-analysis included eight more RCTs [11, 17–22, 26] as compared to those included in the previous systematic review [10] that included both published and unpublished studies. Seven out of the 11 included studies showed a moderate risk in the performance and detection bias; while on considering all the studies together, a low risk of selection bias, attrition bias and reporting bias was observed. Egger’s test did not indicate any obvious publication bias. The included studies were of low to moderate quality.

Previous RCTs showed that additional intervention in the form of plastic wrap in preterm infants may help prevent hypothermia; however, the sample size in these studies was small [18, 19, 26]. Previous systematic review by McCall EM *et al*. showed that plastic wraps were effective in reducing heat loss in infants admission to NICU less than 28 weeks of gestation, but not in infants between 28 to 31 weeks of gestation; and that the plastic wrap significantly reduces the risk of hypothermia on admission to NICU for infants of gestational age < 29 completed weeks [10]. With the emergence of related studies recently, the present meta-analysis focused on determining the efficacy and safety of plastic wrap applied immediately at birth and/or after during NICU for prevention of heat loss in preterm infants, as compared to that achieved with conventional thermal care. Our results suggest a high efficacy of plastic wrap in reducing heat loss both in infants born at less than 28 weeks of gestation, whether admission to NICU or during NICU, as well as in infants born between 28 and 34 weeks of gestation. Moreover, use of plastic wrap was associated with a lower incidence of hypothermia in the preterm infants born at less than 34 weeks of gestation. These results indicate that recommendations should be given to using plastic wrap in preterm infants, as an additional intervention to prevent hypothermia. Sensitivity analysis by the differences in the intervention revealed that different barriers may be the reason for the observed heterogeneity. Further studies should be designed to address the effect of plastic wrap versus plastic bag. Previous systematic review suggested that plastic wrap dose not reduce the risk of death within hospital stay for infants with a gestational age less than 28 completed weeks, which is consistent with the findings reported by Reilly MC *et al*. [11] Our study showed that the application of plastic wrap to preterm infants immediately after birth did not reduce mortality rate for infants of gestational age less than 34 weeks. Only one of the included studies reported data on degrees of severity of hypothermia, and no data demonstrated that the use of plastic wrap can reduce the incidence of severe hypothermia, which is associated with greatest mortality in newborn infants.[11] Hence we cannot conclude at this time that wrapping infants has lifesaving benefits.

Descriptive data on the adverse effects showed that its use was not associated with serious adverse events. The incidence of hyperthermia, the most common adverse effect associated with the use of plastic wrap, was very low, transient and readily reversed after prompt unwrapping.[17, 18, 23, 24]

Some limitations of this meta-analysis need to be acknowledged. Firstly, Most of the included studies had small sample sizes, and very few studies had reported outcomes in relation to the degree of hypothermia. Although there is a need to conduct high quality randomized controlled trials involving a larger number of preterm babies, and reporting outcomes in relation to the degree of hypothermia, as recommended by the World Health Organization [27], in order to draw more meaningful conclusions, performing randomized controlled trials in preterm may be fraught with ethical challenges. Secondly, our study included preterm infants at different locations, with different basic characteristics, and with the use of different barriers to heat loss; all of which could have contributed to the heterogeneity. Thirdly, longer follow-up period is required to assess the impact on mortality and neurodevelopmental outcomes. Moreover, the adverse effects associated with plastic wrap could not be fully evaluated due to the limited availability of data. Lastly, most of the included studies did not evaluate the cost-effectiveness of plastic wrap, which is essential to ascertain its feasibility, particularly in developing countries.[1, 9]

In conclusion, use of plastic wrap can be considered as an effective and safe additional intervention to help prevent hypothermia in preterm infants. However, its cost-effectiveness and long-term impact on mortality needs to be ascertained by conducting well-designed studies with longer follow-up period.

## Supporting Information

S1 PRISMA Checklist PRISMA checklist. (DOC)

S1 Table Characteristics of excluded studies. (DOCX)
