# Supplementary material for: Efficacy and Safety of Plastic Wrap for Prevention of Hypothermia after Birth and during NICU in Preterm Infants: A Systematic Review and Meta-Analysis
Source: PLoS One. 2016 Jun 9;11(6):e0156960. doi: 10.1371/journal.pone.0156960 (PMC4900561; doi:10.1371/journal.pone.0156960)
Supplement: S1 Table — (DOCX) [file pone.0156960.s002.docx]

**Table S1. Characteristics of excluded studies**

| **Study** | **Reason for exclusion** |
| --- | --- |
| Caglar2014[[1](#_ENREF_1)] | This study compared two interventions applied after delivery: vinyl isolation bags or polyethylene wrap. |
| Nuntnarumit2013[[2](#_ENREF_2)] | Intervention was applied polyethylene plastic draping during umbilical catheterization. |
| Belsches2013[[3](#_ENREF_3)] | Participants were term. |
| Simon2011[[4](#_ENREF_4)] | This study compared two interventions applied after delivery: thermal warming mattresses or wrapping in a polyethylene sheet. |
| Kaushal2005[[5](#_ENREF_5)] | Thermal outcome measures were not reported |

**References**

1. Caglar S, Gozen D, Ince Z. Heat loss prevention (help) after birth in preterm infants using vinyl isolation bag or polyethylene wrap. J Obstet Gynecol Neonatal Nurs.2014; 43: 216-223.

2. Nuntnarumit P, Swatesutipun B, Udomsubpayakul U, Thanacharoenpipat P. A randomized controlled trial of plastic drape for prevention of hypothermia during umbilical catheterization. Am J Perinatol.2013; 30: 839-842.

3. Belsches TC, Tilly AE, Miller TR, Kambeyanda RH, Leadford A, Manasyan A, et al. Randomized trial of plastic bags to prevent term neonatal hypothermia in a resource-poor setting. Pediatrics.2013; 132: e656-661.

4. Simon P, Dannaway D, Bright B, Krous L, Wlodaver A, Burks B, et al. Thermal defense of extremely low gestational age newborns during resuscitation: exothermic mattresses vs polyethylene wrap. J Perinatol.2011; 31: 33-37.

5. Kaushal M, Agarwal R, Aggarwal R, Singal A, Upadhyay M, Srinivas V, et al. Cling wrap, an innovative intervention for temperature maintenance and reduction of insensible water loss in very low-birthweight babies nursed under radiant warmers: a randomized, controlled trial. Ann Trop Paediatr.2005; 25: 111-118.
